# Multicolor PEGDA/LCNF Hydrogel in the Presence of Red Cabbage Anthocyanin Extract

**DOI:** 10.3390/gels7040160

**Published:** 2021-09-30

**Authors:** Erlin Arda Safitri, I Putu Mahendra, Anggi Eka Putra, M Alvien Ghifari, Demi Dama Yanti, Yusnaidar Yusnaidar, Bambang Ariwahjoedi, Jose Alberto Mendez

**Affiliations:** 1 Program Studi Kimia, Jurusan Sains, Institut Teknologi Sumatera, Lampung Selatan 35365, Indonesia; erlin.118270066@student.itera.ac.id (E.A.S.); anggi.putra@ki.itera.ac.id (A.E.P.); m.ghifari@ki.itera.ac.id (M.A.G.); demi.damayanti@ki.itera.ac.id (D.D.Y.); 2 Pusat Riset dan Inovasi Sanitasi dan Kesehatan Lingkungan, Institut Teknologi Sumatera, Lampung Selatan 35365, Indonesia; 3 Program Studi Pendidikan Kimia, Fakultas Keguruan dan Ilmu Pendidikan, Universitas Jambi, Jambi 36361, Indonesia; yusnaidar.yusnaidar@unja.ac.id; 4 Program Studi Teknik Material, Jurusan Teknologi Produksi dan Industri, Institut Teknologi Sumatera, Lampung Selatan 35365, Indonesia; bambang.ariwahjoedi@ki.itera.ac.id; 5 Enginyeria Quimica, Universitat de Girona, 17003 Girona, Spain; jalberto.mendez@udg.edu; 6 Laboratori d’Enginyeria Paperera i Materials Polimers, Universitat de Girona, 17003 Girona, Spain

**Keywords:** red cabbage anthocyanin, colorimetric indicator, PEGDA, LCNF

## Abstract

Colorimetric indicator gels were developed by incorporating anthocyanin (AC) obtained from red cabbage into poly (ethylene glycol) diacrylate (PEGDA)-based hydrogel containing lignocellulose nanofiber (LCNF). The PEGDA-based hydrogel was prepared by mixing all of the mentioned components at the specific composition, and the hydrogels were cured under UV light (245 nm) for 1 min. The pH-response, UV absorption, swelling ratio, and mechanical properties of PEGDA/LCNF were determined. It was further found that PEGDA and LCNF mount play an important role in adjusting the mechanical properties of PEGDA/LCNF. In general, the presence of LCNF improved the mechanical properties and swelling ratio of PEGDA. The incorporation of red cabbage anthocyanin into the PEGDA/LCNF film showed multicolor response when specific pH buffers were introduced. Based on the multicolor response of PEGDA/LCNF/CA, this gel film indicator can be developed as a food freshness indicator that focuses on the detection of ammonia and amine compound.

## 1. Introduction

Nowadays, the colorimetric pH indicator has become an interesting topic; it can be developed as a useful product to monitor the freshness of food by applying it as an intelligent packaging or placing it inside the packaging. The colorimetric indicator principle is based on the change in pH of the food. A consumer can quickly determine the food quality without opening the food packaging. The application of this colorimetric indicator can lead to a better life, especially from the aspect of food quality, and could reduce the amount of food waste. With the growth of researcher knowledge, the colorimetric indicator is produced by blending biodegradable materials, mostly polymers, with pH-sensitive dyes. Biodegradable materials are chosen owing to their properties, e.g., environmentally friendly, non-toxic, and decompose in a short amount of time [1,2,3]. These biodegradable materials can be in the form of carbohydrate, protein, and other macromolecules.

However, the use of the colorimetric indicator in film-forming is limited owing to sensitivity. There are several factors that could reduce the sensitivity of the film-based colorimetric indicator, e.g., dissolution of the target due to the presence of water entrapped in the film, as well as temperature and humidity, which also could play that role, due to the storage condition of food [4,5]. These three factors could lead to false detection, which is not suitable for the criteria of intelligent packaging. Hence, current research focuses on the development of colorimetric indicator gels that are prepared by mixing PEGDA and LCNF, and a specific pH-sensitive dye. In the form of hydrogel, the sensitivity of the material improves owing to the presence of pores, and the material has an ability to swell in the presence of water sources. Hydrogel can be described as crosslinked polymer that can swell as a result of the water absorption process [6]. The development of colorimetric indicator gels can also be mentioned as a smart hydrogel that can respond to stimuli (in this case, pH) and adapt to environmental conditions. Hydrogel has been utilized in various fields, e.g., medical purposes, agriculture, wound dressing, and water treatment [7,8,9,10,11,12,13,14,15].

Lignocellulose nanofiber was obtained from the most abundant natural biopolymer, cellulose, which was prepared through TEMPO oxidation and assisted by mechanical processes, e.g., milling and high-pressure homogenizer [16,17,18,19]. Lignocellulose nanofiber was utilized as the nanofiller to enhance the mechanical properties of PEGDA. The properties of PEGDA-based hydrogel can be adjusted by controlling the amount of crosslinking agent and the irradiation time [15]. In several papers, PEGDA has been mentioned as the smart hydrogel material because of this property, and it can also be utilized for tissue engineering purposes [12,14,15]. In this study, a hydrogel indicator was subsequently prepared by incorporating anthocyanins obtained from red cabbage. The hydrogel properties such as pH-response, swelling activity, and mechanical properties were evaluated.

## 2. Results and Discussion

### 2.1. Morphology of LCNF and PEGDA/LCNF

**Figure 1:**
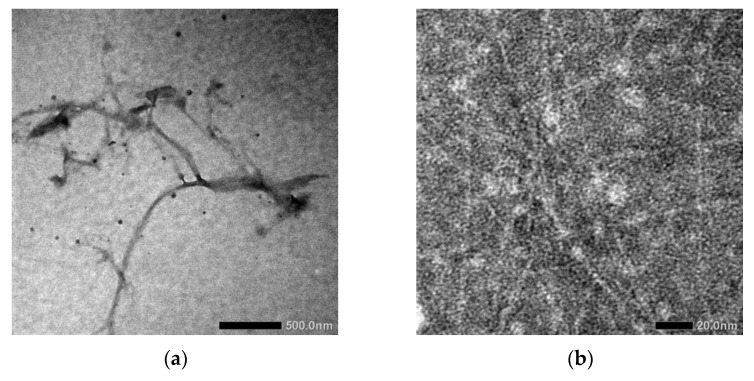
Transmission Electron Microscope (TEM) image of LCNF (**a**) before and (**b**) after high-pressure homogenizer treatment.

Figure 1a,b show the morphology of oxidized fiber after being modified using TEMPO and continued by high-pressure homogenizer, respectively.

**Figure 2:**
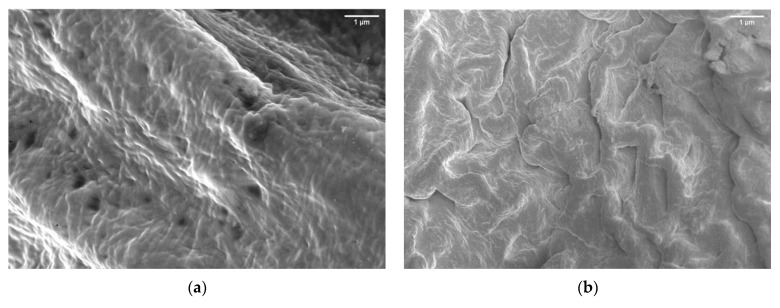
Scanning Electron Microscope (SEM) image of (**a**) PEGDA and (**b**) PEGDA/LCNF.

In Figure 1a, the diameter of LCNF fiber is about 10 nm and the length is over 100 nm. Based on this result, LCNF was still found as a bundle of fibers. The aggregation of fiber to form a bundle of fibers occurred because of the presence of hemicellulose; this phenomenon has been mentioned in several studies [16,20]. Further mechanical treatments are needed to enhance the individualization of LCNF fiber, as shown in Figure 1b. After the LCNF was treated using high-pressure homogenizer, the obtained result showed a better individualized fiber. The use of high-pressure homogenizer promotes the diameter of fiber into less than 5 nm. We can assume that high-pressure homogenizer maximizes the role of the carboxylic group, which is present on the LCNF surface, especially the electrostatic repulsive force between the LCNF fibers [21,22,23,24]. As a result, at the end of this process, a transparent gel-like form was obtained. This was the result of the applied pressure during high-pressure homogenizer, in which water was forced to be adsorbed by the individualized fiber. The surface characteristics of PEGDA/LCNF and PEGDA itself are shown in Figure 2a,b.

The roughness of the PEGDA surface increased after the incorporation of LCNF. This result indicates that LCNF plays an important role in determining the surface characteristics of PEGDA. The previous studies also discussed the increase in surface roughness as a common phenomenon that appeared when a nanofiller was incorporated into the matrix [12,25,26,27]. In this study, LCNF acted as a nanofiller, which not only affected the surface contoured, but also improved the mechanical properties of the matrix. The improvement in PEGDA mechanical properties can be seen in Section 2.4.

### 2.2. Functional Group of PEGDA/LCNF

**Figure 3:**
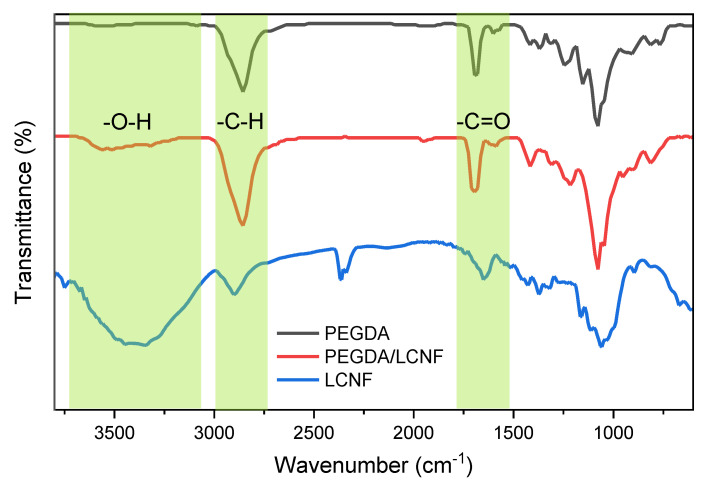
Fourier Transform Infrared (FT-IR) spectra of PEGDA, LCNF, and PEGDA/LCNF.

The Fourier Transform Infrared (FT-IR) spectra of PEGDA, LCNF, and PEGDA/LCNF are shown in Figure 3.

The PEGDA spectrum confirmed the presence of stretching vibration of the hydroxyl group, which appeared at 3400 cm^−1^. Other bands were also found at 2900 and 1730 cm^−1^, which referred to the presence of methyl or methylene and carbonyl group, respectively. The spectrum of LCNF has a different pattern to the PEGDA spectrum. This is because of the different chemical structure between LCNF and PEGDA. LCNF has several distinctive bands, e.g., 3600–3300, 2900–2800, and 1650 cm^−1^, referring to the presence of hydroxyl, C-H, and carboxyl group [16]. The presence of lignin also appeared at 1510–1400 cm^−1^ [28,29]. However, when we analyzed the PEGDA/LCNF spectrum, there was no difference from the PEGDA spectrum. This result indicated that the addition of LCNF into the PEGDA system is only as additive, and there was no chemical interaction/reaction occurring between PEGDA and LCNF. Normally, the presence of a new band or the change in the position of the band can be observed when chemical reactions/interactions occurs.

### 2.3. Rheological Property of PEGDA/LCNF

**Figure 4:**
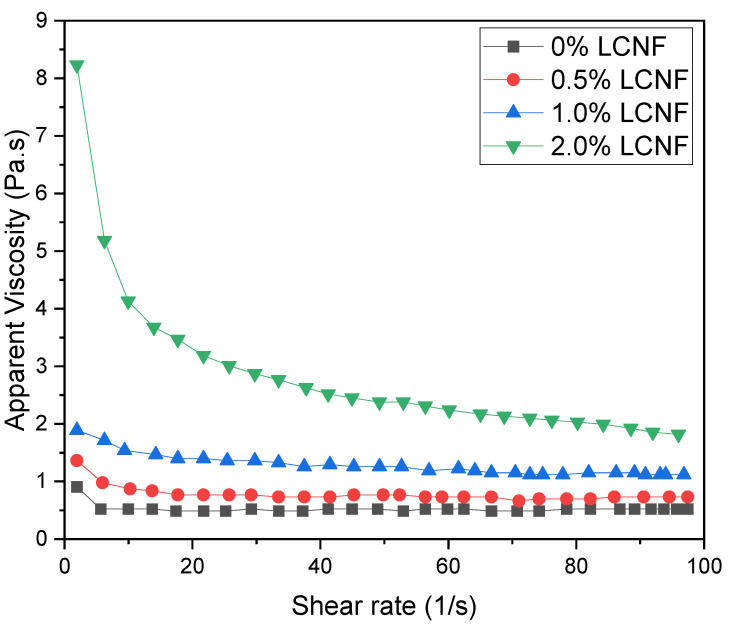
Shear rate vs. apparent viscosity of PEGDA/LCNF.

As mentioned in the hydrogel fabrication, the ratio between PEGDA and LCND varied. The different composition of hydrogel would affect the rheological property of the PEGDA/LCNF hydrogel. The rheology analysis was performed to inspect the apparent viscosity and ability of this mixture to form gel. In Figure 4, the amount of LCNF is able to improve the apparent viscosity of the mixture.

As mentioned in the previous study, nanocellulose can enhance the apparent viscosity and help PEGDA to support the gel structure. For the first two mixtures, there was no significant change in the apparent viscosity as the shear rate increased; however, a shear thinning property can be observed in the third mixture, which consisted of 2.0 *w*/*v*.% of LCNF.

### 2.4. Mechanical Properties of PEGDA/LCNF

**Figure 5:**
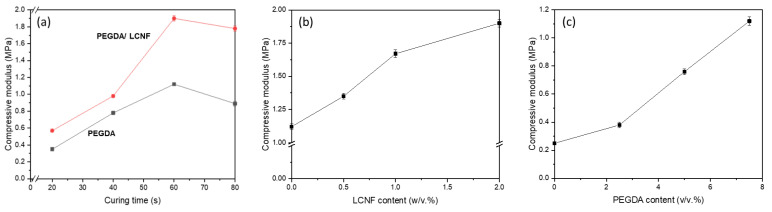
Compression modulus of PEGDA LCNF: (**a**) effect of curing time, (**b**) effect of LCNF content, and (**c**) effect of PEGDA content.

The mechanical properties of the PEGDA/LCNF hydrogel were determined using compression testing. The characteristic of hydrogel was measured by following three aspects, i.e., effect of curing time, effect of LCNF content, and effect of PEGDA content. The effect of curing time was assessed by preparing a specific hydrogel composition, e.g., 7.5 *v*/*v*.% of PEGDA and 2 *w*/*v*.% of LCNF. As in Figure 5a, the addition of LCNF improved the compression modulus of PEGDA up to 69%. Other aspect that could be observed was the effect of curing time. The compression modulus showed an increase in value in both lines (before and after LCNF addition) in line with the curing time. However, these values started to decrease when the curing time was more than 60 s. The crosslinking density could play an important role that can affect the mechanical properties of materials. As mentioned before, the PEGDA hydrogel needs to be cured under UV light sources to initiate the crosslinking process.

Meanwhile, the effect of LCNF content was determined by preparing hydrogel with the following composition: 7.5 *v*/*v*.% of PEGDA and 0.5–2.0 *w*/*v*.% of LCNF, as well as a curing time of 60 s. Figure 5b shows that PEGDA/LCNF has a higher value than PEGDA, and the value increased as the amount of LCNF increased. The compression modulus for the PEGDA hydrogel, which has 0.5, 1.0, and 2.0 *w*/*v*.% of LCNF, was increased by 20.5, 49.1, and 69.6%, respectively. From these two aspects, LCNF plays an important role that can enhance the mechanical properties of the PEGDA hydrogel. As a nanofiller, LCNF has been known as a reinforcing agent that can help improve the mechanical properties of a material. The obtained data are also in line with the results of previous studies that used a derivate of cellulose nanofiber as a nanofiller or reinforcing agent [12,30,31,32,33]. In the third aspect, the hydrogel was prepared with the composition 2.0 *w*/*v*.% of LCNF and 2.5–7.5 *v*/*v*.% of PEGDA, as well as a specific curing time of 60 s. In Figure 5c, the compression modulus was found to increase as the amount of PEGDA increased. This phenomenon can be explained as the higher amount of PEGDA helped the hydrogel have a higher crosslinking component. Based on these three aspects, the conclusion that could be made from this study was that PEGDA was the main component of the hydrogel and acted as a structural material for the 3D structure of the hydrogel, and LCNF, as mentioned before, acted as the reinforcing agent.

### 2.5. Swelling Ratio

**Figure 6:**
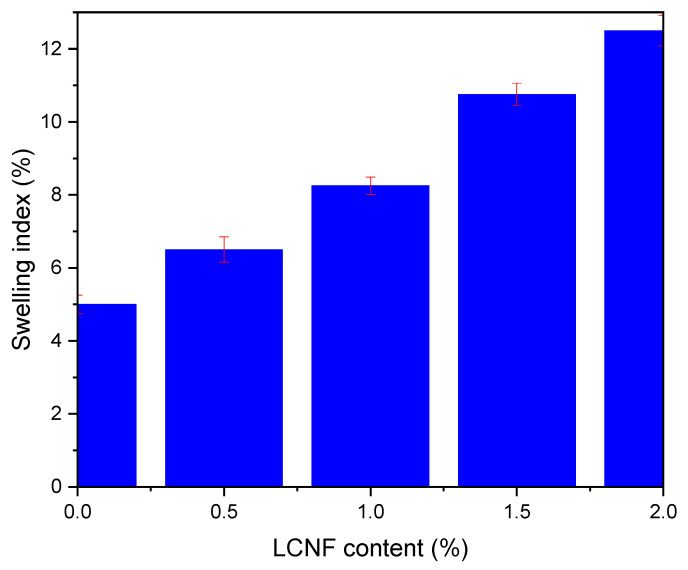
Swelling ratio of PEGDA/LCNF hydrogel (the error bars were determined at *p* ≤ 0.05).

The ability of PEGDA/LCNF to absorb water was determined through a swelling test by calculating the swelling ratio of each hydrogel. The hydrogel was treated by soaking it in distilled water for 24 h. This treatment was performed to obtain the swelling ratio of each hydrogel at the equilibrium condition. The hydrogel used for this characterization was the one with a composition of PEGDA 7.5 *v*/*v*.% and LCNF 0.5–2.0 *w*/*v*% and a curing time of 40 s. The swelling ratio of each hydrogel is shown in Figure 6, which increased as the amount of LCNF increased. In addition, the highest swelling ratio among these prepared hydrogels was at 12.5%. The PEGDA hydrogel only has a 5% swelling ratio. These data can be explained based on the LCNF structure, which is rich in the hydroxyl group on its surface. As common knowledge, the hydroxyl could interact with water through hydrogen bonding. With this swelling ratio value, PEGDA/LCNF could have a good ability as the matrix to entrap the anthocyanin extract, and the sensitivity of the anthocyanin extract in this matrix could be enhanced, as mentioned in a previous study [34].

### 2.6. UV/Vis Spectra

**Figure 7:**
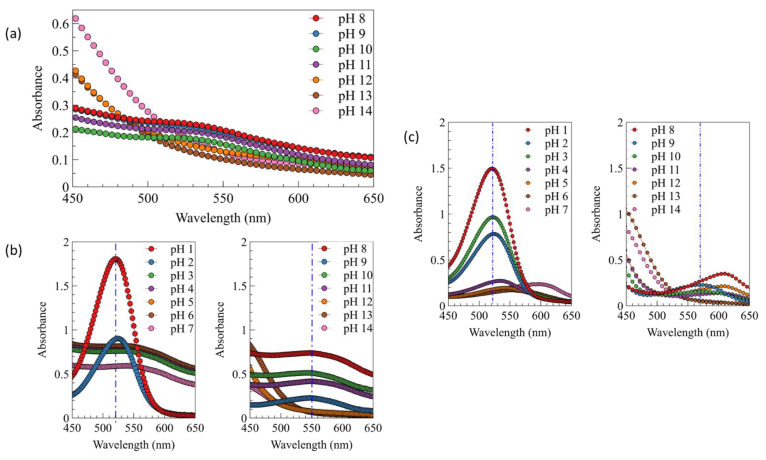
UV/vis spectra of (**a**) fermented anthocyanin extract at different pH (+ NaOH/H_2_SO_4_), (**b**) color of fresh anthocyanin extract at different pH (+ NaOH/H_2_SO_4_), and (**c**) color of fresh anthocyanin extract at different pH (specific buffer).

**Figure 8:**
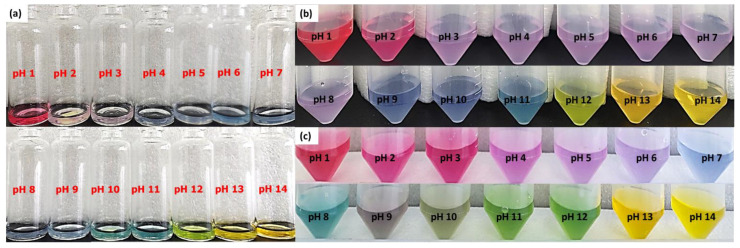
(**a**) Color of fermented anthocyanin extract at different pH (+ NaOH/H_2_SO_4_), (**b**) color of fresh anthocyanin extract at different pH (+ NaOH/H_2_SO_4_), and (**c**) color of fresh anthocyanin extract at different pH (specific buffer).

The color variation of anthocyanin extract was tested using the spectrophotometer UV/vis to determine each spectrum characteristic. The UV/vis spectra in Figure 7 show that the anthocyanin has a different pattern for every pH. This can be described directly from the photo of anthocyanin extract (Figure 8); the color change of anthocyanin extract varied significantly, from red to orangish yellow, when using the fermented extract.

Red color was obtained at pH 1.0, and its intensity slightly decreased to pinkish purple at pH 2.0–3.0. Blue color started to appear at pH 4.0 until 11.0. A different color was finally obtained at pH 12.0 and 13.0–14.0, which was green and orangish yellow. However, when fresh extract was used, a different color range and intensity were obtained. Red color was still obtained at pH 1.0, while the pinkish purple was obtained at pH 2.0 with stronger intensity. The purple color started to appear at pH 3.0 until 8.0. Blue color was only obtained at pH 9.0–10.0, and it turned to greenish blue color at pH 11.0. Yellow color also obtained at pH 12.0, and this color turned to orange at pH 13.0 and 14.0. This wide range of color was only obtained when the fresh extract was used owing to the different pH of extract. The fermented extract has a more acidic pH than the fresh ones; this acidic pH can affect the final pH of solution and, as a result, lead to a different color of solution. A different color was also obtained when the fresh anthocyanin extract was added into a different pH solution prepared using specific buffers. Red pink reddish purple colors were obtained at pH 1.0–3.0. Purple color was obtained at pH 4.0, and the intensity was slightly decreased at pH 5.0 and 6.0. The color turned to blue and bluish green (turquoise) at pH 7.0 and 8.0. Dark bluish green was obtained at pH 9.0; this color slightly turned into green at pH 10.0, and finally turned completely into green at pH 11.0 and 12.0. Then, at pH 13.0 and 14.0, the colors were similar to the previous ones prepared using NaOH solution. The different color between the fresh anthocyanin extract prepared using H_2_SO_4_/NaOH solution and specific buffers could be caused by the presence of the interaction of various ions appearing in the buffer solutions, e.g., potassium, sodium, carbonate, borate, phosphate, and phthalate ions. The presence of these ions could influence the color formation at the specific pH. As mentioned in the previous study, the presence of various cations and anions could act as a co-pigmentation agent, which can enhance the anthocyanin color for each pH of the solution [35,36]. A correlation between Figure 7 and Figure 8 can be utilized to explain the absorbance shift phenomenon from the lower (525 nm) to higher (600 nm) wavelength, because each color of the solution has a specific spectrum pattern. The absorbance peak shifted to the higher wavelength as the pH increased [34,37,38]. This phenomenon is called bathochromic shift, and normally occurs when a structure of flavonoid group—in this case, anthocyanin—changes as a result of pH change or even chemical reaction [37,38].

### 2.7. Color Response

**Figure 9:**
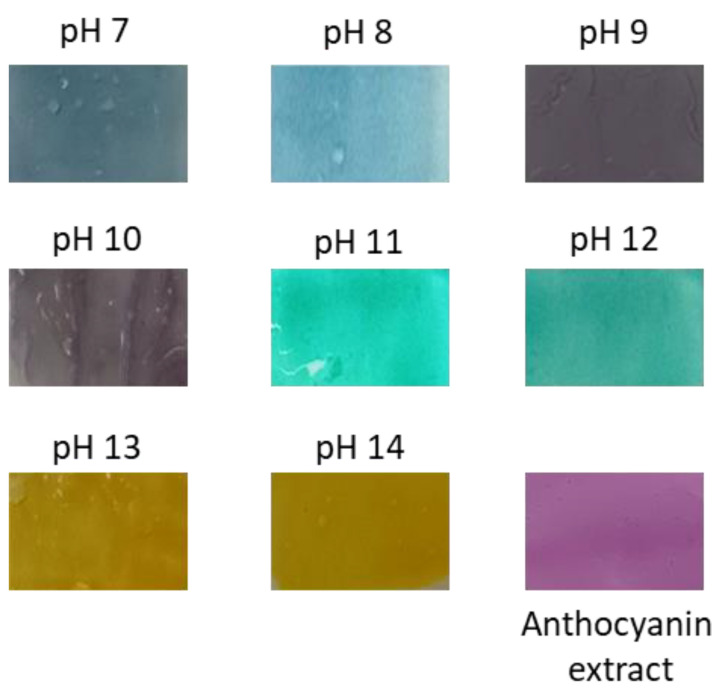
Response color of red cabbage anthocyanin extract embedded in the PEGDA/LCNF film hydrogel.

Based on the produced color of fresh and fermented extract of red cabbage at different pH (1–14), the color response of pH indicator gel was prepared by incorporating the fresh anthocyanin extract. The color response was determined by immersing the gel at different pH buffer solutions (pH 7.0–14.0). This technique was chosen because of the water resistance property of the PEGDA/LCNF hydrogel. The experiment was performed at pH 7.0–14.0 because of the multicolor produced by the fresh extract (Figure 9). The multicolor produced by this extract has a chance to be developed as a food freshness indicator to determine the presence of ammonia and amine compound, which normally started to appear at a pH near 9.0. Based on Figure 8b,c, pH 8.0–10.0 leads to a significant and different visible color compared with the color produced by fermented extract. This significant and different color of anthocyanin extract at different pH solution was due to the help of the PEGDA/LCNF matrix, which can reduce the migration of anthocyanin. Normally, the migration of anthocyanin or dyes from the polymer matrix can influence the sensitivity of polymeric indicator. The crosslinking of the PEGDA structure is the main key that reduces the migration of anthocyanin, and it can be assumed that anthocyanin was entrapped around the PEGDA crosslinked structure. This statement has been mentioned in a related study, not only acting as a matrix to entrap the anthocyanins, and the PEGDA/LCNF materials can also absorb moisture to act as the solid support to prevent false indication. With the moisture-holding ability, the PEGDA/LCNF matrix can enhance the pH indicator sensitivity, owing to the proton transfer phenomenon between the entrapped anthocyanin and pH solution [34].

The *L*, *a*, and *b* values of each color were determined using ImageJ 1.52i (https://imagej.net/software/fiji/index, 29 August 2021), and white paper was used as the control to normalize any factor that influences the *L*, *a*, and *b* values when taking a picture. The L*, a*, and b* values of each produced color at different pH can be seen in Table 1.

The color parameter used in this study is known as CIE LAB, which can be explained as the correlation of each color with the intensity of light. The L value indicates the intensity of light; *L* is categorized as dark when the value is between 0 and 50, and vice versa. Then, the *a* value indicates the redness or greenness; *a* is categorized as red when the value is (+), and vice versa. Finally, the *b* value indicates yellowness or blueness; *b* is categorized as yellow when the value is (+), and vice versa. The correlation among *L*, *a*, and *b* can be explained by taking the color sample obtained in this study.

As mentioned in the previous section, each color has a specific UV/vis spectrum. This is because of the chemical structure stability of anthocyanin extract. The stability of anthocyanin is affected by several factors, e.g., pH, temperature, metal ion, co-pigmentation, and others. The red color at pH 1.0–3.0 is influenced by the presence of flavylium cations [38]. Several studies have mentioned that the colorless carbinol pseudobase should be obtained at pH 3.0–6.0; however, in the current study, purple solutions were obtained instead. This phenomenon could be induced by the presence of mixtures of flavylium cations and its blue tautomer, neutral quinonoidal [38,39]. The intense blue color was obtained at pH 7.0, indicating the presence of neutral quinonoidal species as dominant species in the solution. The green color was significantly changed into green at pH 8.0 owing to the increase in anionic species of hydroxyl; this phenomenon induced the formation of anionic quinonoidal, which has a bluish green color. The intensity of the green color increased as the number of anionic species increased; the green color was only observed until pH 12.0. At the higher pH of 13.0 and 14.0, an orangish yellow color was obtained as a result of the formation of the chalcone structure from anionic quinonoidal species [38,39].

## 3. Conclusions

The controllable hydrogel was successfully prepared by mixing the gel-like texture of LCNF and PEGDA. The compression modulus of PEGDA/LCNF was found to increase up to 69% with the increase in LCNF amount. Not limited to the mechanical property, the viscoelasticity of hydrogel also improved after the addition of LCNF. Based on the current result, the PEGDA/LCNF hydrogel could be utilized as any innovative product; in this study, we tried to evaluate the capability of PEGDA/LCNF as a pH indicator in the form of film. The red cabbage anthocyanin was incorporated into the PEGDA/LCNF film, and a multicolor film was observed when specific buffer was introduced, which is caused by the inherent properties of anthocyanin. Owing to the multicolor result obtained at pH 7.0–14.0 in this study, the PEGDA/LCNF incorporated red cabbage anthocyanin can be proposed or developed as an ammonia indicator for food freshness.

## 4. Materials and Methods

### 4.1. Materials

Ethanol, HCl, NaOH, and PEGDA were purchased from Sigma Aldrich (Singapore). Empty fruit bunches oil palm was obtained from a local plantation in Riau, Indonesia. The red cabbage was supplied from a local market in Jati Agung, Lampung, Indonesia. All chemicals were utilized without any further purification.

### 4.2. Production of Lignocellulose Nanofiber

About 100 g of short EFB fiber was chemically treated using hydrogen peroxide (10 wt.%) under alkaline condition (pH 10) for 2 h at room temperature. The fiber was then washed until the neutral pH of wastewater was obtained. The fiber was passed into a Sproud–Waldron defibrator and PFI mill at a speed of 4000 revolutions. These mechanic treatments were performed to enhance the individualized fiber number. The individualized fiber was then oxidized using TEMPO (0.016 g/g fiber) in the presence of NaBr 0.1 g/g fiber), NaOCl (10 mmol/g fiber) and NaOH to keep the pH reaction at 10. The reaction was stopped after 5 h, and the obtained fiber was washed until the pH of wastewater was neutral. Furthermore, the oxidized fiber (1 g fiber/100 g distillate water) was then treated using a high-pressure homogenizer at 600 bars of pressure for six cycles [16]; this treatment can reduce the fiber size. As the proof, at the end of this process, a transparent gel-like suspension will be obtained.

### 4.3. Hydrogel Fabrication

The PEGDA/LCNF hydrogel was prepared by mixing PEGDA and LCNF at a specific ratio. The photo initiator, lithium phenyl-2,4,6-trimethylbenzoyl, was added into PEGDA (5, 7.5, and 10 *v*/*v*.%) until its final concentration reached 0.05 *w*/*v*.%. Various amounts of LCNF (0.5, 1.0, and 2.0 *w*/*v*.%) were added into the PEGDA solution. The mixture of PEGDA/LCNF was sonicated to ensure the mixture was homogenous. The transparent mixture was then poured into a cylindrical mold (h = 1.0 cm, d = 1.0 cm), and this mixture was irradiated with UV light (365 nm).

### 4.4. Preparation of Dye Solution and pH Sensing

The fresh anthocyanin extract was obtained by macerating 100 g of red cabbage in 100 mL of distilled water. Meanwhile, the fermented anthocyanin extract was obtained by macerating the same amount of red cabbage for 2 weeks at 10 °C. The anthocyanins solution was adjusted to pH 1–14 with the help of a specific buffer. The absorption of each obtained solution was then measured using a UV/vis spectrophotometer. The hydrogels with the highest compressive strength were then prepared as film and immersed in anthocyanin solution for 4 h. The color change of hydrogels was photographed, and the CIE LAB value of each photo was evaluated by ImageJ 1.52i to obtain *L*, *a*, and *b* values. Each sample was evaluated in triplicate. The total color difference (Δ*E*) was calculated by Equation (1):

(1) $$\Delta E=\sqrt{L^{*2}+a^{*2}+b^{*2}}$$

where *L* is the lightness, *a* refers to redness to greenness, and *b* refers to yellowness to blueness of the hydrogel.

### 4.5. Hydrogel Characterization

#### 4.5.1. Fourier Transform Infrared Spectra (FTIR)

The functional group of the hydrogel was determined using FT-IR spectrophotometer 8201PC Shimadzu, which was performed in the wavenumber range from 4000 to 500 cm^−1^ at 4 cm^−1^ of resolution. The sample was pulverized and mixed with KBr.

#### 4.5.2. Scanning Electron Microscopy (SEM)

The surface morphology of each sample was carried out under scanning electron microscope (SEM) HITACHI TM4000. The sample was gold-sputtered using a sputter coater to enhance the conductivity of sample and placed on the sample holder.

#### 4.5.3. Swelling Behavior

The dried hydrogels (*W_i_*) were weighed and immersed in deionized water at room temperature. Samples were taken out of the water at time intervals, and the excess surface water was removed gently with blotting paper. Next, the weight of swollen hydrogels (*W_t_*) was measured. The swelling ratio (%) was calculated using Equation (2):

(2) $$S=\frac{W_{t}}{W_{i}}\times 100\%$$

where *W_i_* is the dried hydrogel’s weight and *W_t_* is the swollen hydrogel’s weight at time *t*. All measurements were conducted in triplicate.

#### 4.5.4. Rheology

Rheology properties of each sample were carried out in a shear rate range from 0.1 to 100 s^−1^, and temperature was set at 25 °C. The measurement was performed using Rheometer Physica MCR 302 (Anton-Paar, Ashland, VA, USA).

#### 4.5.5. Mechanical Properties

The unconfined compression technique was used to determine the mechanical properties of samples. The measurement was carried out using universal testing machine (Instron, model 5944) at a speed rate of 0.5 mm/min.

### 4.6. Statistical Analysis

The statistical analysis was performed to determine the significant differences of L*, a*, and b* values in the range of pH 7.0–14.0. The results were reported in the format of the mean ± standard deviation (SD). One-way ANOVA with Tukey test was used for multiple comparison between data. The level of confidence was that of *p* ≤ 0.05.

## Figures and Tables

**Table 1:** L*, a*, and b* values of each produced color at different pH based on Figure 9.

pH	L*	a*	b*	E
7	42.80 ± 1.63 ^A^	10.33 ± 1.25 ^A^	−11.30 ± 0.90 ^A^	45.49 ± 1.47 ^A^
8	41.98 ± 0.48 ^A^	14.65 ± 0.26 ^B^	−12.73 ± 0.20 ^B^	46.25 ± 0.49 ^A^
9	32.46 ± 0.01 ^B^	4.12 ± 0.02 ^C^	−19.04 ± 0.03 ^C^	37.86 ± 0.02 ^B^
10	30.79 ± 0.06 ^B^	4.76 ± 0.26 ^D^	−17.52 ± 0.20 ^D^	35.75 ± 0.12 ^C^
11	28.62 ± 0.13 ^C^	−5.77 ± 0.12 ^E^	−12.55 ± 0.25 ^E^	31.78 ± 0.12 ^D^
12	45.45 ± 0.12 ^D^	−13.62 ± 0.19 ^F^	45.35 ± 0.04 ^F^	65.63 ± 0.10 ^E^
13	49.27 ± 0.08 ^E^	7.55 ± 0.24 ^G^	48.28 ± 0.01 ^G^	69.40 ± 0.05 ^F^
14	56.25 ± 0.34 ^F^	−1.69 ± 0.22 ^H^	55.27 ± 0.16 ^H^	78.88 ± 0.31 ^G^

^A–H^ Different superscripts in the same parameter indicate significance differences (*p* ≤ 0.05).
